# Surgery Extraordinary

**Published:** 1887-04

**Authors:** 


### SURGERY EXTRAORDINARY.

### Cutting Through a Man's Side to get at Diseased Organs.

Over a year and a half ago, there was admitted to the male surgical ward a young man, Abraham Bradway, aged twenty-two, and to all outward appearances, of sound health; to the physician in charge he said he was a consumptive, having for many years, as he supposed, been a victim to the ravages of that disease. His physician, he alleged, had diagnosed his affliction as phthisis pulmonalis, and had treated him accordingly, but despite his efforts, no change in his system was noted.

In addition to this, the tibia of his right leg was found to be much decayed — so much, in fact, that the physicians at the hospital were at first disposed to look upon the entire lower part of the limb as dead bone, there being no flesh at all on that portion of his leg between the knee and the ankle joints. This condition had existed, the young man said, for over four years, and all treatment he had received proved fruitless of any good results.

Shortly after Bradway's admission to the hospital he began to grow noticably worse, and in a few weeks was so emaciated that his own friends failed to recognize him, and his life was dispaired of. He had fallen in weight from 162 to 91 pounds, and had refused to eat anything, saying that the sight of food nauseated him.

From the then existing symptoms, one of the physicians was led to believe that instead of the patient being a consumptive, he was afflicted with empyema, or a formation of pus in one of the cavities of the body. Further investigation proved that he had been correct in his diagnosis, and preparations were at once begun to locate the formation. By means of a probe it was found to be in the space between the serous membrane sac around one of the lungs and the lung itself. A tube was introduced through the cartilage between the seventh and eighth ribs and the sac punctured. For several days there was a steady flow of pus through the tube, and the patient professed to be growing better. It was not long, however, before the state of affairs was an exceedingly delicate and dangerous one, and owing to the complications existing it was impossible to use the probe and tube as before. The sac had refilled with fast-forming pus, and as the space between the serous membrane and the lung was occupied, the heart of the young man was pushed out of position towards the right side of the body, and the lung so greatly compressed as to entirely stop respiration. The situation was, indeed, alarming, and caused much excitement among the physicians, who had manifested much interest in the case. As a dernier resort it was finally decided to reach the seat of the trouble by cutting the side of the body.

The young man was informed of the intentions of the physicians and readily gave his consent. He was placed under the influence of the most powerful anaesthetics, owing to his extremely weakened condition, and several inches of the seventh and eighth ribs cut away, leaving the terribly distended lungs in full view.

No one of the internal organs was in its proper place, the heart was pushed full up to the right side of the body by the diseased lungs and already showed traces of a similar affection. The lungs themselves had been swollen so that they occupied nearly the entire thorax, and in a few days respiration would have been totally suspended.

As soon as the young man was able to stand the operation, the pus sac was again punctured and the entire contents drained therefrom. Over a gallon of pus and bruised blood was drawn from his lungs and the sac.

Thoroughly cleansed, the opening in his side was then covered with a silver plate and the skin brought together over it. A very light diet was ordered, and for several weeks the patient hovered between life and death. At the end of a month, however, he began to improve, much to the satisfaction of the attending physician, and last week was able to leave his bed. He now weighs 140 pounds, eats heartily, and is of great assistance around the wards. Amputation of his leg was not found necessary, as it began to grow better as the empyema disappeared—*Louisville Courier Journal.*

